# Value of preoperative enteroscopic carbon nanoparticle labeling in guiding laparoscopic resection of Meckel’s diverticulum

**DOI:** 10.1055/a-2612-3215

**Published:** 2025-06-26

**Authors:** Chen Wu, Lingyun Wang, Xiangyu Liu, Chunlin Wang, Jing Cao

**Affiliations:** 1 70570 The Second School of Clinical Medicine, Southern Medical University, Guangzhou, China; 2 117947 Department of Gastroenterology, Jining No.1 Peopleʼs Hospital, Jining, China; 3 117947 Department of Emergency Abdominal Surgery, Jining No.1 Peopleʼs Hospital, Jining, China

Meckel’s diverticulum is a congenital digestive tract malformation, with a prevalence of 0.3% to 2.9% in the general population 1 2 3 . Only 15% of patients with Meckel’s diverticulum are symptomatic and preoperative diagnosis with gastrointestinal endoscopy is limited 3 . Enteroscopy provides precise anatomical guidance for minimally invasive surgery. Herein, we report two rare cases of patients presenting with hematochezia. In both cases, enteroscopy revealed the Meckel’s diverticulum, and carbon nanoparticle labeling was subsequently performed. The lesions were resected via laparoscopic surgery guided by carbon nanoparticle labeling ( Video 1 ).

Value of preoperative enteroscopic carbon nanoparticle labeling in guiding laparoscopic resection of Meckel’s diverticulum.Video 1

**Patient 1:** A 20-year-old man presented with a 4-day history of hematochezia. Previous gastrointestinal endoscopy failed to identify the bleeding source. Subsequent enteroscopy revealed a diverticulum with a narrow opening, located 200 cm proximal to the ileocecal valve. Submucosal injection of carbon nanoparticles was performed around the lesion for marking. Then, laparoscopic exploration guided by carbon nanoparticle labeling confirmed the diverticulum, measuring 4 × 1.2 cm, and segmental small-bowel resection was performed. Histopathology confirmed Meckel’s diverticulum with normal ileal wall ( Fig. 1 ).

**Fig. 1:**
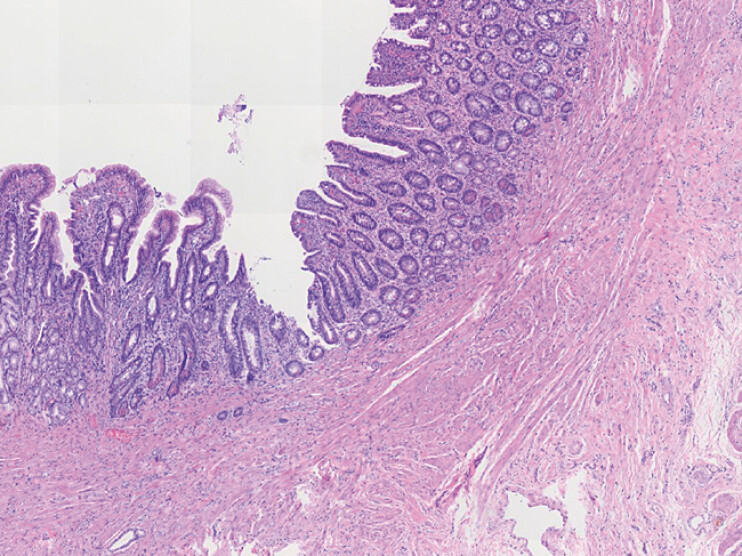
Microscopic appearance. A loupe view of the resected Meckel’s diverticulum showed normal ileal wall with well-formed villi and the muscularis propria (hematoxylin and eosin ×4).

**Patient 2:** A 17-year-old man was admitted with recurrent hematochezia for over 1 year and recurrence for 3 days. Previous colonoscopy failed to identify the bleeding source. Subsequent enteroscopy found a giant diverticulum with the blind end of the diverticulum showing irregular mucosal protrusions ( Fig. 2 ). Narrow-band imaging showed heterotopic gastric mucosa characteristics ( Fig. 3 ). A tortuous submucosal artery with visible pulsation was observed adjacent to the lesion. The lesion was marked using carbon nanoparticles and a preliminary diagnosis of Meckel’s diverticulum was established. Then, the patient was transferred for laparoscopic surgical intervention. Laparoscopic exploration revealed the giant diverticulum in the distal ileum, approximately 100 cm proximal to the ileocecal valve ( Fig. 4 ). Histopathology confirmed Meckel’s diverticulum containing heterotopic gastric mucosa ( Fig. 5 ).

**Fig. 2:**
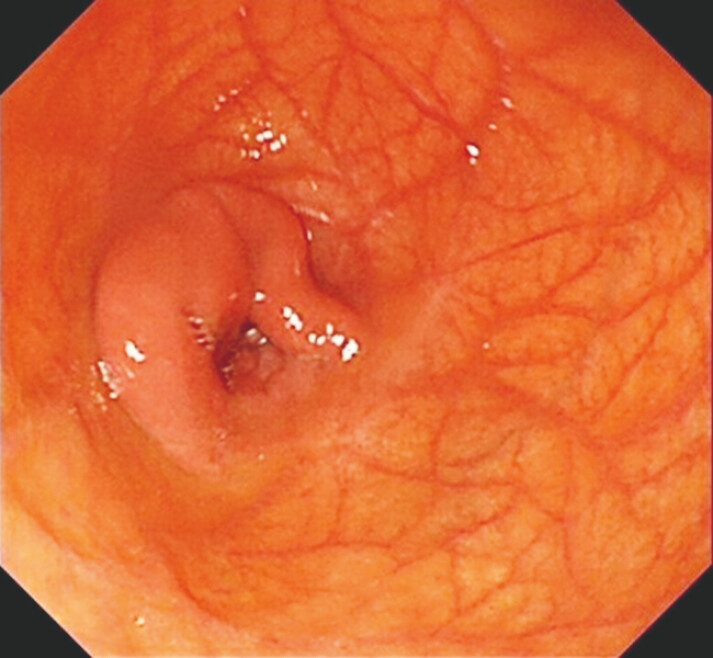
The enteroscopy found a giant diverticulum with the blind end of the diverticulum showing irregular mucosal protrusions.

**Fig. 3:**
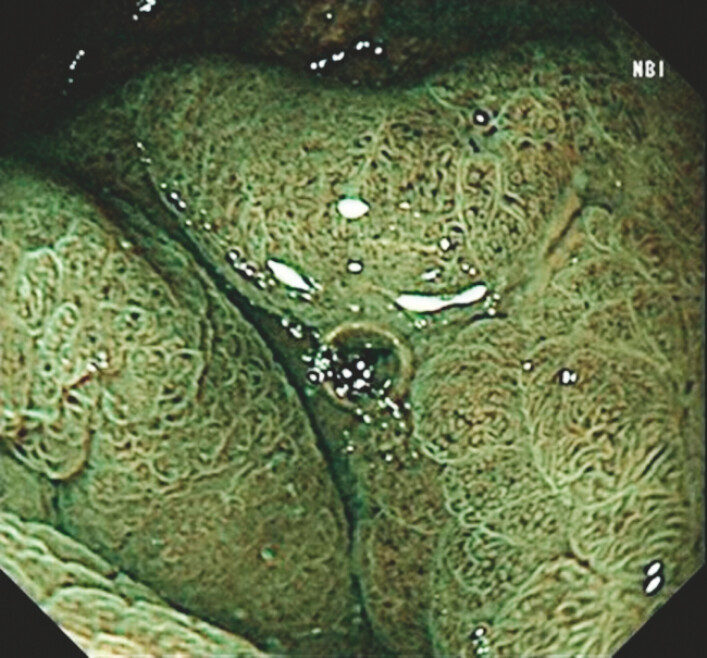
Narrow-band imaging showed heterotopic gastric mucosa characteristics.

**Fig. 4:**
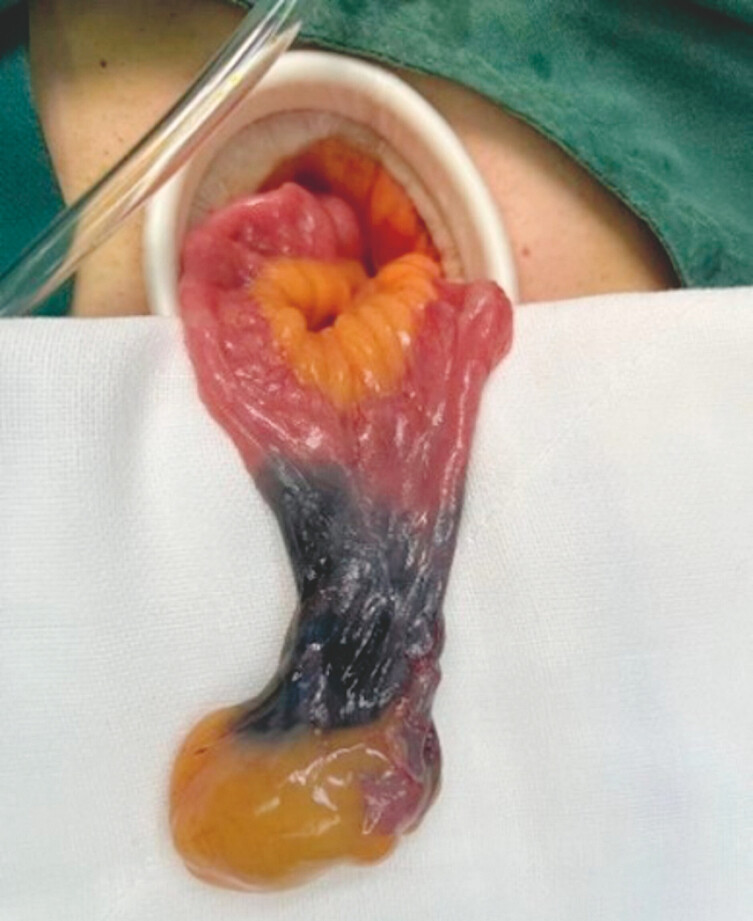
Laparoscopic surgery revealed Meckel’s diverticulum in the ileum, approximately 100 cm proximal to the ileocecal valve.

**Fig. 5:**
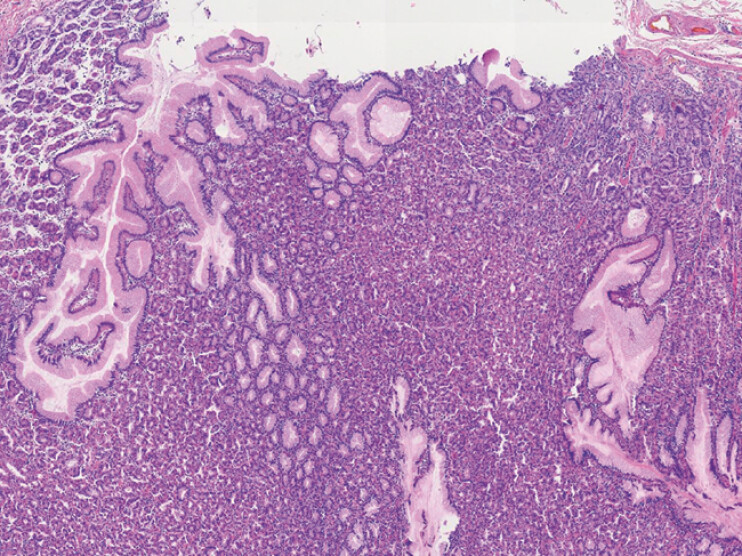
Microscopic findings. A loupe view of the heterotopic gastric mucosa of Meckel’s diverticulum (hematoxylin and eosin ×4).

Enteroscopy with carbon nanoparticle labeling provides precise anatomical guidance for minimally invasive surgery.

Endoscopy_UCTN_Code_CCL_1AC_2AF

## Notes

Endoscopy E-Videos https://eref.thieme.de/e-videos
*E-Videos* is an open access online section of the journal *Endoscopy* , reporting on interesting cases and new techniques in gastroenterological endoscopy. All papers include a high-quality video and are published with a Creative Commons CC-BY license. Endoscopy E-Videos qualify for HINARI discounts and waivers and eligibility is automatically checked during the submission process. We grant 100% waivers to articles whose corresponding authors are based in Group A countries and 50% waivers to those who are based in Group B countries as classified by Research4Life (see: https://www.research4life.org/access/eligibility/ ). This section has its own submission website at https://mc.manuscriptcentral.com/e-videos .
